# Physiological provocation compared to acetazolamide in the assessment of cerebral hemodynamics: a case report

**DOI:** 10.1186/s13550-024-01154-x

**Published:** 2024-10-02

**Authors:** Alexander Cuculiza Henriksen, Gerda Krog Thomsen, Gitte M. Knudsen, Trine Stavngaard, Sverre Rosenbaum, Lisbeth Marner

**Affiliations:** 1 Department of Clinical Physiology and Nuclear Medicine, Copenhagen University Hospital Bispebjerg and Frederiksberg, Copenhagen, Denmark; 2 Department of Radiology, Copenhagen University Hospital Rigshospitalet, Copenhagen, Denmark; 3 Department of Clinical Medicine, Copenhagen University, Copenhagen, Denmark; 4 Neurobiology Research Unit, Copenhagen University Hospital Rigshospitalet, Copenhagen, Denmark; 5 Department of Neurology, Copenhagen University Hospital Bispebjerg and Frederiksberg, Copenhagen, Denmark

**Keywords:** Cerebral perfusion, Limb shaking TIA, Cerebrovascular reactivity, Hemodynamic failure, Cerebral perfusion imaging, Positron emission tomography, Single photon emission computed tomography, Brain, Stroke

## Abstract

**Background:**

Severe large vessel disease may lead to cerebral hemodynamic failure that critically impairs cerebral blood flow (CBF) regulation elevating the risk of ischemic events. Assessment of the condition is often based on changes in CBF during vasodilatation; however, pharmacologically induced vasodilation does not reflect the physiological condition during an ischemic event caused by hemodynamic failure. We compared a [^15^O]H_2_O PET brain scan during vasodilation to a [^99m^Tc]HMPAO SPECT brain scan during an ongoing transient ischemic attack (TIA).

**Case presentation:**

A single patient presenting with limb-shaking TIA underwent CT, Digital Subtraction Angiography, and two different modalities of cerebral perfusion scans: [^15^O]H_2_O PET and [^99m^Tc]HMPAO SPECT. Acetazolamide was used in the PET scan to induce vasodilatation, and during the SPECT scan physiological stress, standing up rapidly, was used to induce limb-shaking TIA. CT-angiography and Digital Subtraction Angiography revealed an occlusion in the distal part of the right A2 segment of the anterior cerebral artery, with a corresponding infarction in the watershed area. Collaterals supplied the main vascular territory of the anterior cerebral artery. During rest, neither perfusion modalities demonstrated reduced perfusion outside of the ischemic core. However, we found a pronounced difference between the PET utilizing acetazolamide and the SPECT during the TIA. The PET scan demonstrated relative hypoperfusion in vascular territory supplied by collaterals, while the area around the ischemic core was not affected. Contrary, the SPECT had only minor relative hypoperfusion in the collateral-supplied area, whereas the watershed area proximal to the infarct core had pronounced relative hypoperfusion.

**Conclusions:**

The observed discrepancy in compromised areas during physiological provocation compared to pharmacological induced vasodilation questions the use of an unphysiological stressor for assessment of cerebrovascular hemodynamics. A physiological provocation test may achieve more clinically relevant evaluation.

## Background

Severe large vessel disease in the cervicocerebral arteries can lead to reduced post stenotic perfusion pressure, resulting in decreased cerebral blood flow (CBF). This reduction in CBF prompts compensatory changes in the cerebrovascular system, aimed at maintaining CBF and ensuring adequate oxygen delivery. Such hemodynamic compromise, commonly referred to as hemodynamic failure, is recognized as a risk factor for ischemic stroke and cognitive dysfunction [1, 2] CBF can be measured using several of Cerebral Perfusion Imaging (CPI) techniques [3]. To identify cerebral hemodynamic failure due to flow-limiting stenosis, CPI is performed in a resting and vasodilated state, the latter typically induced by intravenous acetazolamide or inhalation of a hypercapnic gas (e.g. 5%) [4–6].

Some of the CPI techniques, mainly [^15^O]H_2_O PET, allow for CBF quantification and calculation of the relative increase in CBF during vasodilatation, known as Cerebrovascular Reactivity (CVR). Low CVR has been demonstrated to be a strong prognostic measure for stroke [7], although we recently showed that CBF during vasodilation has an even higher prognostic value than CVR [8].

Conversely, the SPECT radiotracer technetium-99m hexamethyl propylenamine oxime ([^99m^Tc]HMPAO) is utilized by clinicians and researchers to assess regional differences in CBF [9]. [^99m^Tc]HMPAO is trapped in the cells, providing a temporal 'snapshot' of the CBF at the moment of injection [10], and subsequent imaging 30–60 min later.

Despite methodological differences, both [^15^O]H_2_O PET and [^99m^Tc]HMPAO SPECT have been part of clinical routine, with [^15^O]H_2_O PET being the reference standard [11], due to its superior quantitative analysis. In advanced large vessel disease, first existing collateral vessels are recruited. Then, these vessels remodel and grow (arteriogenesis), and new collateral vessels may form (angiogenesis) to maintain blood flow to the brain [12]. During pharmacologically induced vasodilation, the vascular resistance in collaterals is higher than in the intrinsic vessels. As a result, blood flow diverts from the collateral-dependent areas towards unaffected areas with lower vascular resistance, potentially causing low or even negative CVR measurements in the collateral-dependent areas—a phenomenon known as “steal” [13]. While the steal phenomenon reflects a state of cerebral hyperemia due to vasodilatation, ischemic events and symptoms are associated with hypoperfusion. Therefore, understanding hemodynamics during hypoperfusion could be more clinically relevant. Further, randomized trials of extracranial-intracranial bypass have failed to establish evidence for the prevention of secondary ischemic stroke possibly partly due to a lack of robust identification of patients at risk [14, 15]. A reliable diagnosis of hemodynamic failure can only be achieved multimodally, and is dependent on an assessment of the clinical manifestations. A notable example of such a symptom is Limb-Shaking Transient Ischemic Attack (TIA), which is characterized by non-rhythmic limb movements and is considered pathognomonic for hemodynamic failure [16]. Limb-shaking TIA is typically triggered by factors such as positional changes, hyperventilation, or postprandial states, which temporarily reduce cerebral perfusion pressure, leading to decreased CBF [17]. Our study aimed to examine the CBF distribution during physiological provocation in a patient with inducible Limb-shaking TIA due to a stenosis in the A2 segment of the anterior cerebral artery.

### Case presentation

The enrolled patient, a 71-year-old man, was included from Department of Neurology, Copenhagen University Hospital Bispebjerg and Frederiksberg. The [99^m^Tc]HMPAO SPECT scans was performed as part of a study protocol, while the other imaging was conducted as part of the clinical evaluation. This study was approved by the Regional Committee of Health Research Ethics for the Capital Region of Denmark (H-21049132), and written participation consent was obtained after receiving oral and written information.

The patient presented with a series of stereotyped neurological symptoms that were consistent with limb-shaking TIA. The patient reported incidents of limb shaking in the left leg only (corresponding to occlusion of the right Anterior Cerebral Artery—see below) when mobilizing from sitting to standing. The patient's medical history included diabetes mellitus type II, hypercholesterolemia, hypertension, myocardial infarction, an ejection fraction of 35%, obesity, a biological aortic valve prosthesis, and an implantable cardioverter-defibrillator. The patient was a previous smoker.

#### Imaging

1. *CT* Initial structural assessment. No MRI was performed due to metal implants.
2. *Digital subtraction angiography (DSA)* A single 5F catheter was sequentially repositioned in the internal and external carotid artery on both side, as well as the left vertebral artery. For each catheter position, following contrast injection with Visipaque (270 mg I/mL), anteroposterior and lateral views were systematically acquired. Additionally, oblique views were obtained for the right internal carotid artery. A total of 95 ml Visipaque was used.
3. *[*^*15*^*O]H*_*2*_*O PET* Participant underwent a total of four PET scans in a Biograph Vision Quadra (Siemens Healthineers, Erlangen, Germany) after four injections of approximately 400 MBq [^15^O]H_2_O: two scans at rest and two during vasodilatation induced by infusion of 1500 mg acetazolamide (Diamox®). The scanning protocol with the input function was extracted from the descending aorta as previously been described [18]. CBF was quantified with a one-tissue compartment model (Zhou GRRSC) using PMOD software (Pmod Technologies LLC, Switzerland). The results of the kinetic modeling from two rest scans and the two stress scans were averaged to reduce noise. CVR was calculated using the following formula: CVR = (CBF_Diamox_ – CBF_rest_)/CBF_rest_.
4. *[*^*99m*^*Tc]HMPAO SPECT* The study incorporated [^99m^Tc]HMPAO SPECT to assess cerebrovascular parameters under two conditions. For Limb-shaking TIA provocation, the participant consumed a fat and carbohydrate-rich meal approximately 30-60 minutes prior. Valsalva was initiated while the patient was seated, followed by a rapid postural change and an intravenous injection of approximately 900 MBq [^99m^Tc]HMPAO 3 s after experiencing limb-shaking TIA symptoms. A neurologist (SR) closely monitored the limb-shaking TIA symptoms that lasted 10–20 s. Scanning was performed 40 min after radioligand injection with an AnyScan Trio SPECT scanner (Mediso, Hungary) as previously described [19]. After a 2-week interval, an unprovoked, resting [^99m^Tc]HMPAO SPECT was conducted. Eyes were open during both injections. The scans were analyzed using Scenium tool, Syngo.via (Siemens Healthineers, Germany).

DSA, PET, and SPECT were conducted within three months, and approximately a year after the initial CT scan.

**Fig. 1:**
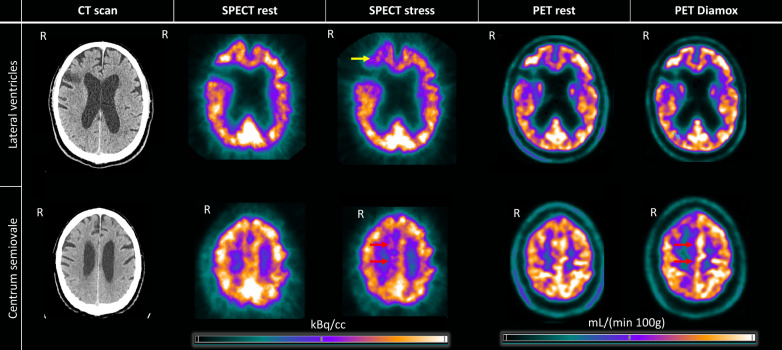
CT scan and CPI maps from the SPECT (kBq/cc) and PET (mL/(min∙100 g)) scans during rest and physiological stress or acetazolamide (Diamox®) induced vasodilation. The PET CBF maps show increased perfusion from around 40 (mL/(min∙100 g) in rest to around 70 (mL/(min∙100 g) during acetazolamide. However, the images have been visually scaled to approximate the global mean, facilitating comparison. The top row shows sections at the level of the lateral ventricles, while the bottom row shows sections at the level of the centrum semiovale. The yellow arrow indicates an area affected only on the SPECT scan, while the red arrows show affection of the mesial frontal lobe, most notably observed on the PET scan. R: right

*CT* The CT scan revealed sequelae of an ischemic infarct corresponding to the watershed area in the premotor area on the right side (Fig. 1).

**Fig. 2:**
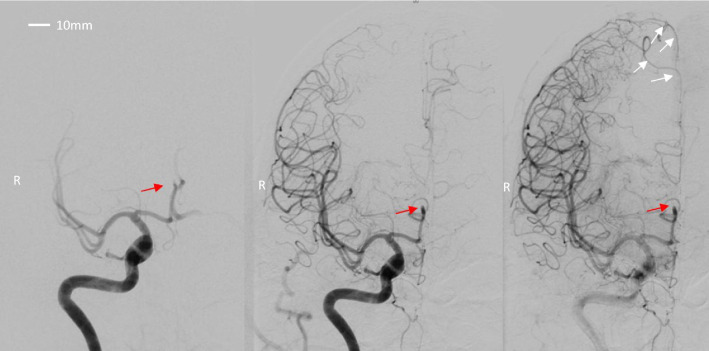
Digital Subtraction Angiography from the right carotid artery in three different phases, demonstrating the A2 occlusion (red arrow) along with the collaterals from the Middle Cerebral Artery (white arrows). R: right

*DSA* DSA imaging demonstrated a right-sided occlusion in anterior cerebral artery at the A2 segment, presumably at the bifurcation of the pericallosal and callosomarginal arteries. Clear collaterals were seen from both the Middle Cerebral Artery and the leptomeningeal vessels to the supply area of ​​the affected anterior cerebral artery. Blood flow appeared preserved but delayed, as visually interpreted by the interventional radiologist, indicating slowed perfusion. The area with infarct on the CT was seen with several small disorganized artery structures. (Fig. 2).

**Fig. 3:**
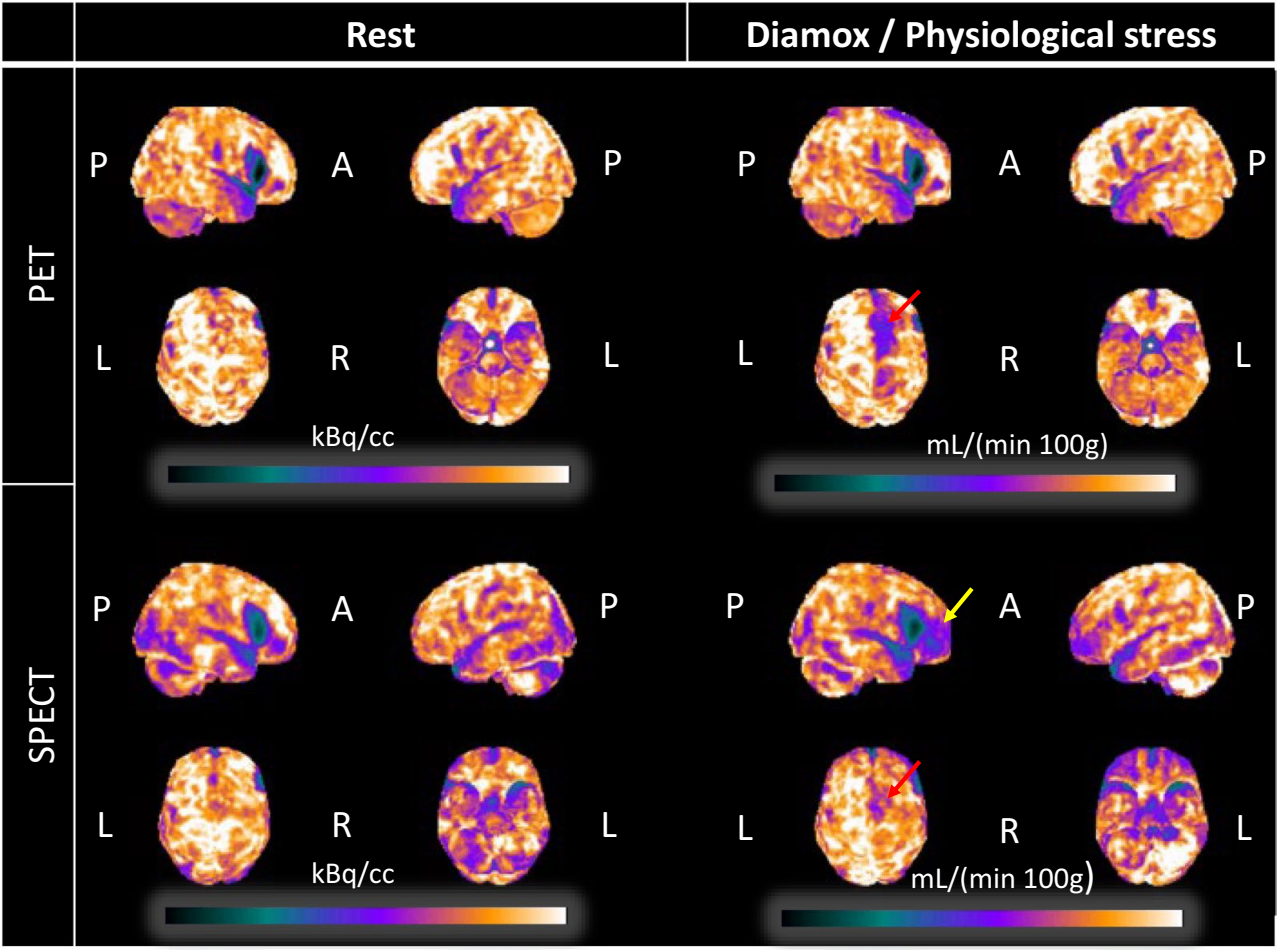
Surface projections of the CBF and SPECT (kBq/cc) and PET (mL/(min∙100 g)) scan during rest and physiological stress or Diamox. The images have been visually scaled to approximate the global mean, facilitating comparison. The yellow arrow indicates an area affected only on the SPECT scan, while the red arrows show a reduction in the mesial frontal lobe, most notably observed on the PET scan. P: posterior, A: anterior, L: left, R: right

**Fig. 4:**
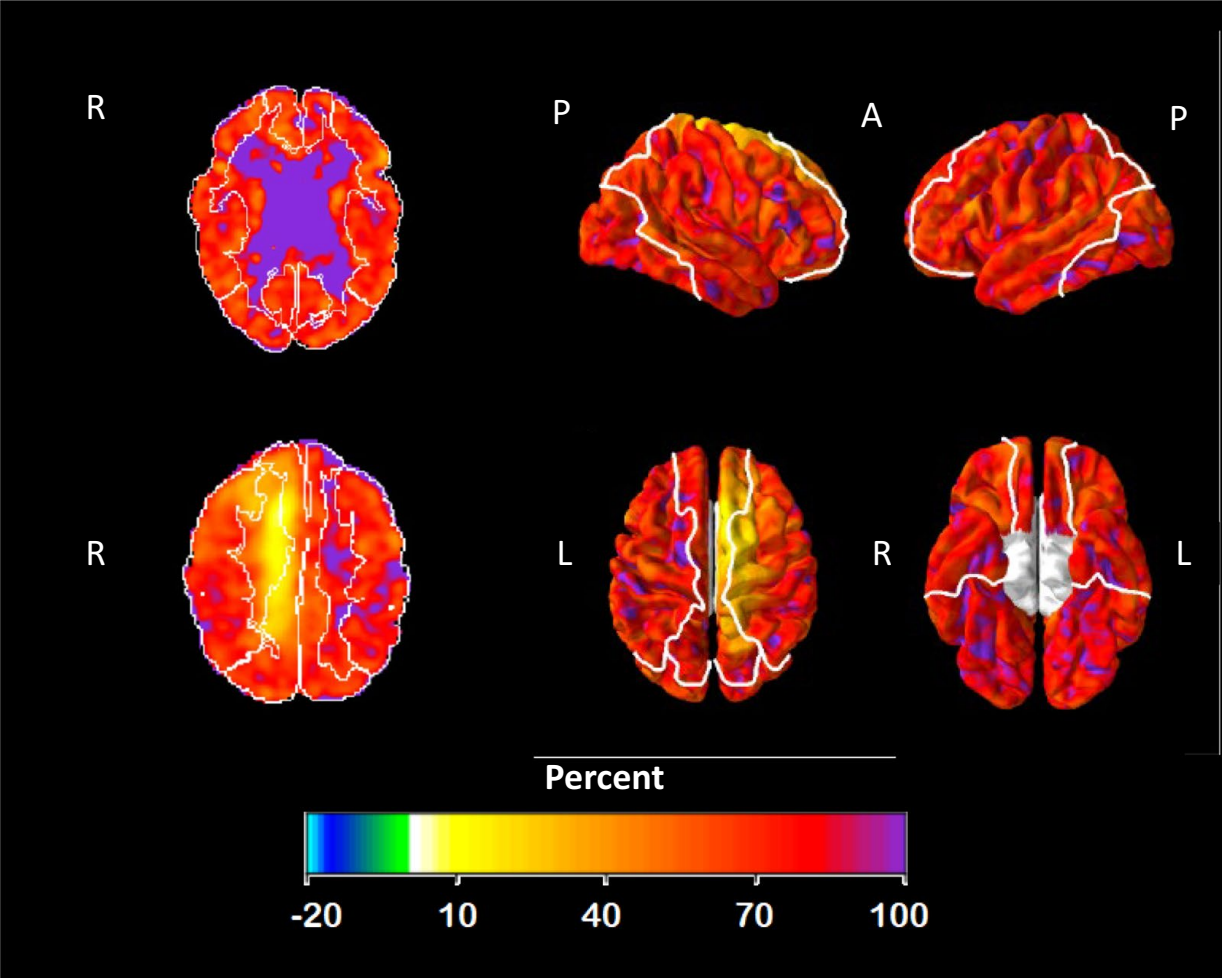
PET CVR maps. On the left, images show the CVR maps at the level of the lateral ventricles (top) and the centrum semiovale (bottom). On the right are the CVR surface projections. The examination demonstrates a low CVR of 0–5% in the right mesial frontal lobe corresponding to the distal ACA segment, while the region surrounding the infarction core at the level of the lateral ventricles appears normal

*[*^*15*^*O]H*_*2*_*O PET* Apart from the watershed area with the known infarction, the baseline scan was normal (Figs. 1, 3 and 4). The scan demonstrated a reduced CVR of 0–5% in the mesial frontoparietal region, corresponding to the distal part of the callosomarginal artery i.e. downstream of the occluded A2. The CVR during vasodilatation surrounding the premotor infarction in the right frontal lobe was normal (Fig. 4).

*[*^*99m*^*Tc]HMPAO SPECT imaging* Upon standing, the patient experienced mild symptoms consistent with limb-shaking TIA in the left leg. The baseline [^99m^Tc]HMPAO SPECT scan showed no uptake corresponding to the known watershed infarction and slightly reduced uptake in the mesial frontoparietal region on the same side. The provocation test demonstrated pronounced relative hypoperfusion in the frontal cortex in proximity to the watershed infarction, whereas the mesial frontoparietal region, corresponding to the supply of the A2 segment exhibited only a minor hypoperfusion These findings corresponded with the watershed infarction region seen on the CT scan (Fig. 1).

#### Comparative analysis

The CT scan demonstrated an infarct in the right premotor area compatible with a watershed infarct (Fig. 1). A right-sided occlusion in the A2 segment was visible on the DSA, and the examination depicted formation of collateral blood supply from both the middle cerebral artery and leptomeningeal vessels to the mesial frontoparietal area (Fig. 2). This area also showed a reduced CVR in the range of 0–5% at the [^15^O]H_2_O PET scan (Fig. 4). While the [^15^O]H_2_O PET scan showed preserved CVR in areas surrounding the watershed infarctions, the [^99m^Tc]HMPAO SPECT, during physical provocation, demonstrated hypoperfusion in the area, mainly anterior to the watershed infarction (Figs. 1, 3 and 4).

## Conclusions

Our objective was to evaluate regional CBF during physiological provocation in a patient with hemodynamic failure due to cerebrovascular large vessel disease. Surprisingly, whereas physiological provocation resulted in focal hypoperfusion in proximity to the watershed infarct as measured with SPECT; the same area displayed normal vascular reactivity during vasodilatation using [^15^O]H_2_O PET (Fig. 4). DSA had uncovered that the patient had an A2 occlusion and that the mesial frontoparietal region was supplied by collaterals (Fig. 2). Whereas the mesial frontoparietal area was affected during vasodilation on the [^15^O]H_2_O PET scan, the region anterior to the watershed infarct appeared unaffected (Figs. 1 and 3). In contrast, [^99m^Tc]HMPAO SPECT scan using physiological provocation demonstrated a relatively mild reduction of perfusion in the mesial frontoparietal region compared to a more pronounced hypoperfusion anterior to the watershed infarction (Figs. 1 and 3).

It was particularly noteworthy that the regions which seemed compromised during vasodilatation on [^15^O]H_2_O PET scan were well supplied by collateral pathways. During a potent global vasodilation stimulus such as acetazolamide, blood is diverted to the healthy middle cerebral artery territory due to a global decreased vascular resistance resulting in delayed and reduced CBF through the collaterals. Thus, the reduction in the frontoparietal region is more pronounced during acetazolamide as compared to physiological stress that preserves the vascular resistance.

While CVR based on vasodilation remains a valuable prognostic tool, we identified a scenario where its diagnostic scope may be limited. Importantly, methods relying on vasodilatation could highlight non-critical areas and overlook critical areas, which might influence treatment options, such as revascularization strategies—whose role in managing cerebral hypoperfusion is still debatable [14]. Indeed, new infarcts due to hemodynamic failure are often seen in the watershed area.

Several limitations should, however, be taken into account. The present data is only based on a single subject as non-operated patients with ongoing frequent limb-shaking TIA are rare. More cases should be evaluated to understand the difference between vasodilation and physiological provocation. It should be noted that both the imaging modality as well as the physiological stimulus have been changed and we cannot know for sure which caused the observed differences between scans. The symptoms of LS-TIA manifested 10–20 s. We injected a tracer immediately as the subject rose; however, the delay in tracer arrival to the brain means it probably did not reach cerebral circulation during the symptomatic phase. Therefore, while it is likely that the brain experienced hypoperfusion for a duration exceeding the observable symptoms, direct assessment of cerebral conditions during LS-TIA is not possible. Further, SPECT does not allow for proper quantitative assessment of the difference between rest and physiological provocation test. Previous research has revealed discrepancies between [^99m^Tc]HMPAO SPECT and [^15^O]H_2_O PET, where PET detected impaired CVR in areas not identified by SPECT. However, investigations exploring the opposite scenario—areas detected by SPECT but missed by PET—are notably absent. These methodological differences could contribute to the variances observed in our results, emphasizing the need for a balanced evaluation of both imaging modalities to fully understand their diagnostic capabilities [20]. Although the CPI scans were conducted with three months apart, we believe the impact is minimal due to the clinical presentation.

To summarize, we recommend a nuanced approach to interpreting CVR, augmented by alternative diagnostic methodologies like DSA, transcranial Doppler ultrasound, and potentially physiological tests with perfusion imaging. Utilizing a multi-modal diagnostic strategy offers a more comprehensive view of cerebral health; enhancing the quality of care and risk assessment for these patients.

## Data Availability

All data generated during this study are included in this published article.

## Abbreviations

CBF: Cerebral blood flow
CPI: Cerebral perfusion imaging
CVR: Cerebrovascular reactivity
DSA: Digital subtraction angiography
[^99m^Tc]HMPAO: Technetium-99m hexamethyl propylenamine oxime
TIA: Transient ischemic attach
